# Survivin and NAIP in Human Benign Prostatic Hyperplasia: Protective Role of the Association of *Serenoa repens*, Lycopene and Selenium from the Randomized Clinical Study

**DOI:** 10.3390/ijms18030680

**Published:** 2017-03-22

**Authors:** Giuseppe Morgia, Antonio Micali, Mariagrazia Rinaldi, Natasha Irrera, Herbert Marini, Domenico Puzzolo, Antonina Pisani, Salvatore Privitera, Giorgio I. Russo, Sebastiano Cimino, Antonio Ieni, Vincenzo Trichilo, Domenica Altavilla, Francesco Squadrito, Letteria Minutoli

**Affiliations:** 1 Department of Urology, Polyclinic Hospital, University of Catania, 95124 Catania, Italy; gmorgia@policlinico.unict.it (G.M.); salvoprivi82@gmail.com (S.P.); giorgioivan@virgilio.it (G.I.R.); ciminonello@hotmail.com (S.C.); 2 Department of Biomedical Sciences, University of Messina, 98125 Messina, Italy; amicali@unime.it (A.M.); puzzolo@unime.it (D.P.); apisani@unime.it (A.P.); daltavilla@unime.it (D.A.); 3 Department of Clinical and Experimental Medicine, University of Messina, AOU Policlinico “G. Martino”, Torre Biologica, 5th Floor, Via C. Valeria, Gazzi, 98125 Messina, Italy; mrinaldi@unime.it (M.R.); nirrera@unime.it (N.I.); hrmarini@unime.it (H.M.); vtrichilo@unime.it (V.T.); fsquadrito@unime.it (F.S.); 4 Department of Human Pathology, University of Messina, 98125 Messina, Italy; aieni@unime.it

**Keywords:** BPH, inhibitors of apoptosis proteins, Ser-Se-Ly association

## Abstract

Benign prostatic hyperplasia (BPH) treatment includes the apoptosis machinery modulation through the direct inhibition of caspase cascade. We previously demonstrated that *Serenoa repens* (Ser) with lycopene (Ly) and selenium (Se) reawakened apoptosis by reducing survivin and neuronal apoptosis inhibitory protein (NAIP) levels in rats. The aim of this study was to evaluate the effectiveness of Ser-Se-Ly association on survivin and NAIP expression in BPH patients. Ninety patients with lower urinary tract symptoms (LUTS) due to clinical BPH were included in this randomized, double-blind, placebo-controlled trial. Participants were randomly assigned to receive placebo (Group BPH + placebo, *n* = 45) or Ser-Se-Ly association (Group BPH + Ser-Se-Ly; *n* = 45) for 3 months. At time 0, all patients underwent prostatic biopsies. After 3 months of treatment, they underwent prostatic re-biopsy and specimens were collected for molecular, morphological, and immunohistochemical analysis. After 3 months, survivin and NAIP were significantly decreased, while caspase-3 was significantly increased in BPH patients treated with Ser-Se-Ly when compared with the other group. In BPH patients treated with Ser-Se-Ly for 3 months, the glandular epithelium was formed by a single layer of cuboidal cells. PSA showed high immunoexpression in all BPH patients and a focal positivity in Ser-Se-Ly treated patients after 3 months. Evident prostate specific membrane antigen (PSMA) immunoexpression was shown in all BPH patients, while no positivity was present after Ser-Se-Ly administration. Ser-Se-Ly proved to be effective in promoting apoptosis in BPH patients.

## 1. Introduction

Benign prostatic hyperplasia (BPH) is an age-related disorder of the prostate gland [1]. Clinically evident BPH is a significant health problem mainly for older men, owing to the negative impacts on their quality of life. BPH is characterized by excessive growth of the prostate, which can compress the urethra and/or grow into the bladder, thus inducing lower urinary tract symptoms (LUTS) [1]. LUTS are usually divided into obstructive and irritative: the former are more common, even if the latter are more troublesome and have a greater impact on the lives of patients [2]. Structurally, the increase of prostate volume is triggered by a progressive growth of both the epithelium and the fibromuscolar stroma [3], in the transition zone of the gland at first [4].

BPH pathogenesis still remains incomplete, even if recent evidence demonstrated the role of some pathological conditions, such as chronic inflammation [5,6], abnormal wound repair, deregulation of circulating hormonal levels [7], altered expression of cytokines and chemokines (IL-8 in particular) [8], and disturbance of immune surveillance and recognition [3,7]. Its progression may be the effect of multiple factors, among which changes in epithelial-stromal interactions [9] and in molecular associations between androgens, estrogens, growth factors, and neurotransmitters [6] are included.

In the development of BPH, a damage of the delicate equilibrium between mitogenic and cell death-inducing stimuli, particularly important in prostate tumorigenesis, is not completely clarified [10].

Programmed cell death comprises many molecular steps, culminating in the clearance of impaired and altered cells, while at the same time avoiding the leaking of deleterious substances into the surrounding tissues [11,12]. The characterization of the apoptotic pattern of prostate hyperplastic cells may also yield useful information concerning their possible malignant transformation, thus providing novel therapeutic targets [10].

An emerging role in this setting is played by the inhibitors of apoptosis proteins (IAPs), which block programmed cell death under different stimuli [13]. IAPs modulate apoptosis by inhibiting caspases [14]. In human prostate, an increased IAPs expression was demonstrated in BPH, prostatic intraepithelial neoplasia, and cancer [15].

Recently, we have demonstrated that, in experimental BPH, IAPs proteins—such as survivin and neuronal apoptosis inhibitory protein (NAIP)—are increased, and that phytotherapeutic supplements markedly reduced their expression [16]. In particular, *Serenoa repens* (Ser), derived from Saw Palmetto, is frequently used alone [17]. Ser is also associated with other phytotherapeutic compounds, such as Lycopene (Ly), a carotenoid; and Selenium (Se), an essential trace element. In this way, a therapeutic activity has been obtained in BPH [18]. Furthermore, triple Ser-Se-Ly combination was shown to be more effective than Ser alone in the recovery of apoptosis, reducing prostatic growth [19,20]. In light of this background, we aimed to evaluate the effects of the association of Ser-Se-Ly on IAPs in men with BPH.

## 2. Results

### 2.1. Clinical Features

At enrollment, the mean age of the men was 65 years (range: 55–79); mean prostate specific antigen (PSA) was 6.1 ng/mL (range: 4.3–8.7); mean Qmax was 11.8 (range: 4–15); mean prostate volume was 43 cc (range: 17–60); mean International Prostatic Symptoms Score (IPSS) was 19 (range: 12–35); and mean postvoid residual (PVR) was 50 cc (range: 0–140). Significant differences at baseline between groups regarding age, IPSS, and PSA were not observed (Table 1). After 3 months, no differences were observed in group A patients (data not shown), while a significant decrease of IPSS (mean difference: −2.0 [1.0–3.0]; *p* < 0.05), but not for PSA (mean difference: −0.5 [0.2–0.7]; *p* = 0.4) and Qmax (mean difference: 1.5 [0.5–2.0]; *p* = 0.3) was present in group B patients.

### 2.2. Ser-Se-Ly Decreases Survivin and NAIP Expression

**Figure 1:**
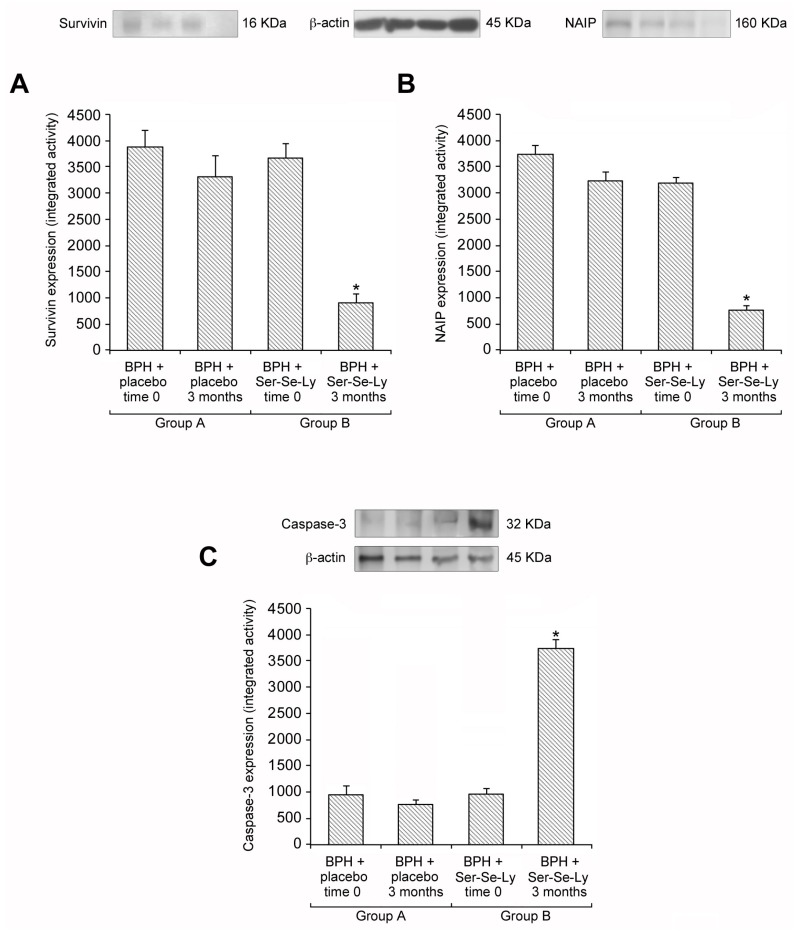
Representative Western Blot analysis of survivin (**A**); neuronal apoptosis inhibitory protein (NAIP) (**B**); and caspase-3 (**C**) expression in the prostate from benign prostatic hyperplasia (BPH) patients at time 0 and at 3 months; and from Ser-Se-Ly treated BPH patients at time 0 and at 3 months. * *p* < 0.05 vs. BPH patients at time 0 and at 3 months, and Ser-Se-Ly treated BPH patients at time 0. Bars represent the mean ± SE of 90 patients.

In the prostatic tissue of BPH patients, at both time 0 and after 3 months following placebo administration (group A), high expressions of survivin and NAIP were detected (Figure 1A,B). Similar data were also observed in BPH patients challenged with Ser-Se-Ly at time 0 (group B) (Figure 1A,B); while, after 3 months of treatment (group B), a significantly reduced survivin and NAIP expression was evident (Figure 1A,B).

### 2.3. Ser-Se-Ly Increases Caspase-3 Expression

In the prostatic tissue of BPH patients, at both time 0 and after 3 months following placebo administration (group A), low expression of caspase-3 was detected (Figure 1C). Similar data were also observed in BPH patients challenged with Ser-Se-Ly at time 0 (Figure 1C). After 3 months of treatment with Ser-Se-Ly (group B), caspase-3 expression was significantly increased (Figure 1C).

### 2.4. Histological Evaluation

**Figure 2:**
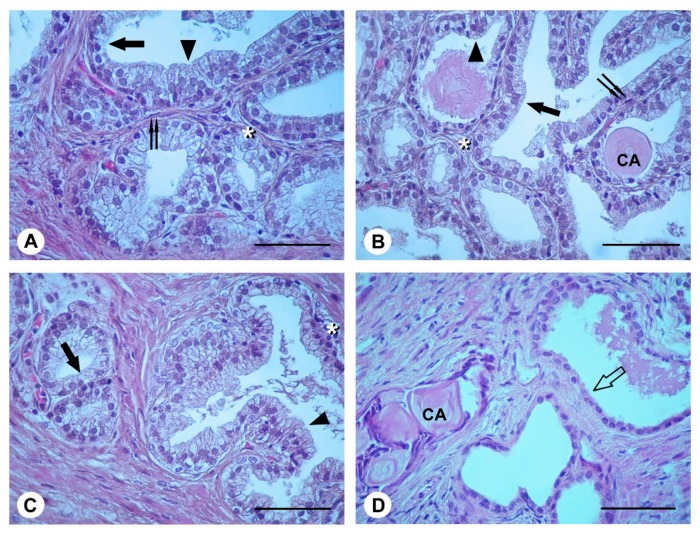
Histological patterns of the prostate from BPH patients at time 0; from BPH patients at 3 months; from Ser-Se-Ly treated BPH patients at time 0; and from Ser-Se-Ly treated BPH patients at 3 months; stained with hematoxylin and eosin (H&E). (**A**–**C**) In BPH patients at time 0 (**A**), in BPH patients at 3 months (**B**); and in Ser-Se-Ly treated BPH patients at time 0 (**C**); glandular structures are lined by simple, tall columnar (arrow) or pseudo-stratified (arrowhead) epithelium, formed by cells with euchromatic nuclei and weakly stained cytoplasm. Smaller basal cells with dark nuclei (*) are also present. On their outer surface, the glands show a thin, discontinuous layer of flat cells with elongated nuclei (double arrow). In the glandular lumen, occasional corpora amylacea (CA) are present; (**D**) In Ser-Se-Ly treated BPH patients at 3 months, the glandular epithelium is formed by a single layer of cuboidal cells with euchromatic nuclei (empty arrow). Some corpora amylacea (CA) are present. (Scale bar: 50 µm).

In the prostate from BPH patients at time 0, from BPH patients at 3 months (group A), and from Ser-Se-Ly treated BPH patients at time 0 (group B), observed with light microscopy (Figure 2A–C), glandular structures were lined by tall columnar or pseudo-stratified epithelium, formed by cells with euchromatic nuclei and weakly stained cytoplasm. Smaller cells with dark nuclei were present in the basal part of the epithelium. On their outer surface, the glands showed a thin, discontinuous layer of flat cells with elongated nuclei. In the glandular lumen, occasional corpora amylacea were present. In BPH patients treated with Ser-Se-Ly for 3 months (group B) (Figure 2D), the glandular epithelium was formed by a single layer of cuboidal cells with euchromatic nuclei. Some corpora amylacea were also present.

### 2.5. Ser-Se-Ly Decreases PSA Immunoexpression

**Figure 3:**
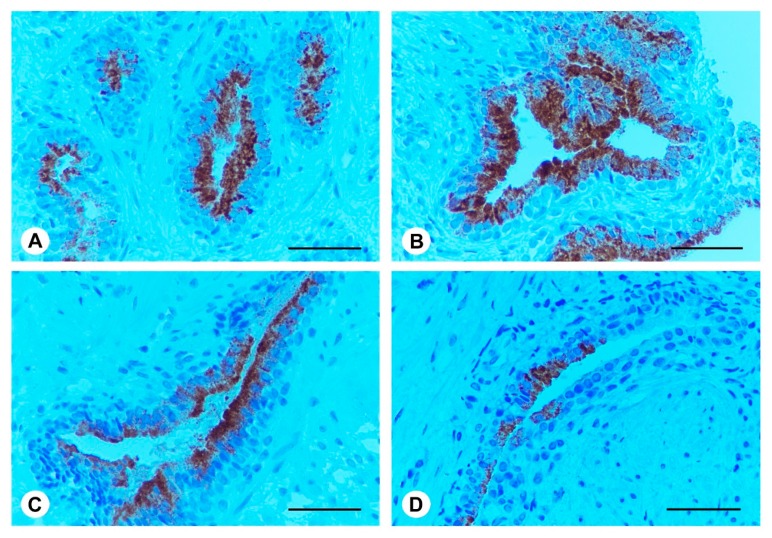
Prostate specific antigen (PSA) immunohistochemistry in the prostate from BPH patients at time 0; from BPH patients at 3 months; from Ser-Se-Ly treated BPH patients at time 0; and from Ser-Se-Ly treated BPH patients at 3 months. (**A**,**C**) In BPH patients at time 0 and in BPH patients treated with Ser-Se-Ly at time 0, uniform PSA immunoexpression is present in the apical portion of the epithelial cells cytoplasm. Basal and stromal cells are negative; (**B**) Similar high immunoexpression is evident in BPH patients after 3 months; (**D**) After 3 months of Ser-Se-Ly administration, PSA immunoexpression is focal in the pseudostratified epithelium, while basal cells are unreactive. (Scale bar: 50 µm).

In the prostate of BPH patients at both time 0 and after 3 months (group A), uniform PSA immunoexpression was present in the apical part of the epithelial cells, while basal and stromal cells were negative (Figure 3A,B). Similar high immunoexpression was evident in Ser-Se-Ly treated BPH patients at time 0 (group B) (Figure 3C). After 3 months of Ser-Se-Ly administration (group B), PSA immunoexpression was focal in the epithelium, while basal cells were unreactive (Figure 3D).

### 2.6. Ser-Se-Ly Decreases in Prostate Specific Membrane Antigen (PSMA) Immunoexpression

**Figure 4:**
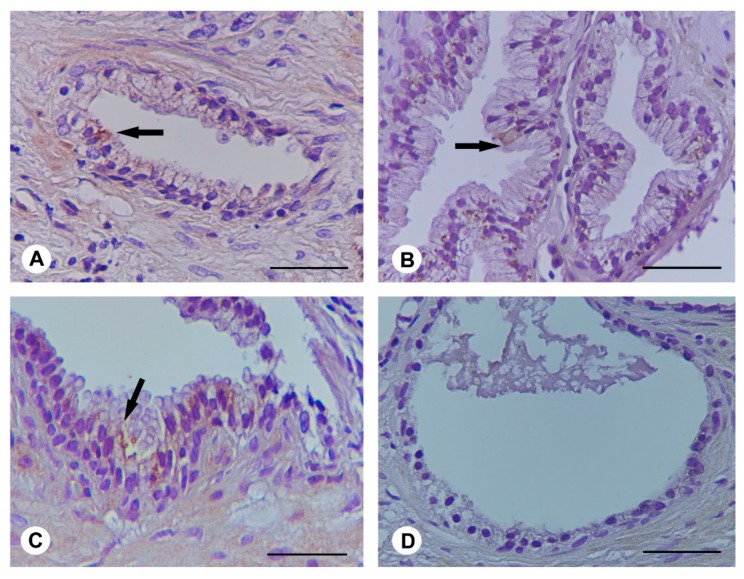
Prostate specific membrane antigen (PSMA) immunohistochemistry in the prostate from BPH patients at time 0; from BPH patients at 3 months; from Ser-Se-Ly treated BPH patients at time 0; and from Ser-Se-Ly treated BPH patients at 3 months. (**A**,**C**) In BPH subjects at both time 0 and in BPH patients treated with Ser-Se-Ly at time 0, PSMA evident immunoexpression is present (arrow); (**B**) Similar evident immunoexpression is observed in BPH patients after 3 months (arrow); (**D**) After 3 months of Ser-Se-Ly administration, no immunoexpression for PSMA is present. (Scale bar: 50 µm).

In the prostatic tissue of BPH patients at both time 0 and after 3 months (group A), evident immunoexpression for PSMA was observed in the tall columnar or pseudo-stratified epithelium (Figure 4A,B). Similar data were observed in BPH patients challenged with Ser-Se-Ly at time 0 (group B) (Figure 4C). After Ser-Se-Ly administration (group B), no immunoexpression for PSMA in the single layer of cuboidal cells was present (Figure 4D).

## 3. Discussion

Ser has been used for the improvement of symptoms in patients with LUTS/BPH. However, although current guidelines cannot make any specific recommendation on phytotherapy for the treatment of male LUTS because of product heterogeneity, limited regulatory framework, and the methodological limitations of published trials, recently a short-term study on the combination of plant extracts with tamsulosin was published with promising results [21]. We have recently demonstrated that the combined treatment with Ser-Se-Ly and tamsulosin was more effective than single therapies (Ser-Se-Ly or tamsulosin) in improving IPSS and increasing Qmax in patients with LUTS at 12 months [20]. The combined treatment with Ser and tamsulosin was more effective than tamsulosin monotherapy in reducing storage symptoms; but IPSS, voiding subscore, QoL, Qmax, PVR, PSA, and prostate volume were unchanged [20].

The triple combination of Ser-Se-Ly has been used to potentiate their therapeutic activity in prostatic diseases [16]. The rationale of this triple therapeutic approach could be the enhanced apoptotic and anti-inflammatory activity.

Recently, it was shown that BPH is associated with inflammation [22]. In fact, data from the study REDUCE (Reduction by Dutasteride of Prostate Cancer Events) evidenced the association between BPH and chronic prostate inflammation in 78.3% of patients who underwent prostate biopsy [5]. Moreover, the occurrence of chronic inflammation has been associated with a greater risk of disease progression and different responses to medical therapy [8,23]. On these bases, the therapeutic use of Ser-Se-Ly—which shows an inhibitory effect on the expression of COX-2, 5-LOX and iNOS [23], and on the mean PSA value [18]—has a role in counteracting inflammation in BPH; thus confirming the role of the association of phytotherapeutics (Ser-Se-Ly) in decreasing the production of inflammatory mediators [24].

In a previous clinical trial (SELECT), the positive impact of Se plus vitamin E on human prostate carcinoma cell lines was demonstrated, showing an evident connection between Bax and Bcl-2 modulation and induction of apoptosis [25]. A similar mechanism able to induce the modulation of the apoptosis machinery is shown, for the first time in the present paper, using a triple combination of Ser-Se-Ly in BPH patients. In these subjects, the molecular analysis of NAIP, survivin and caspase-3 revealed that these apoptosis modulators are crucial in prostate homeostasis. As a matter of fact, these results confirm previous published data by our laboratory in an experimental animal model of BPH [16].

NAIP was originally identified while investigating childhood muscular atrophy, and was also related to spinal muscular dystrophy [26,27]; it has been suggested that NAIP triggers apoptosis by inhibition of the executioner caspases-3 and -7 [27]. Survivin could have an important role in the negative regulation of apoptosis [28]; however the mechanism by which it controls programmed cell death has not been completely clarified [27], even if it was proposed that survivin could modulate the executioner caspases-3 [29,30]. However, recent findings suggest that IAPs have a much broader spectrum of biological activity, as they might also act on inflammatory and innate immune signaling pathways [31].

Indeed, after 3 months of treatment, Ser-Se-Ly significantly reduced survivin and NAIP expression. This feature clearly indicates that IAPs molecular signaling pathway is a potential target to limit the prostatic growth in BPH patients. In this context, the increase of caspase-3 levels after 3 months of Ser-Se-Ly treatment provides further evidence that the phytotherapeutic supplement specifically modulates the apoptotic molecular cascade.

PSA is a 33 KDa glycoprotein consisting of chymotrypsin-like proteins, produced by normal, hyperplastic, and malignant prostate epithelia [31]. PSMA is a 100 KDa type II membrane protein expressed in all forms of prostate tissues, including prostate carcinoma [32,33]. Therefore, we assessed these markers in our BPH patients to evaluate the possible progression of disease. As expected, our results strongly suggest that Ser-Se-Ly treatment is useful in modulating the prostate growth. In fact, immunohistochemical analysis revealed reduced immunoreactivity for PSA and no immunoreactivity for PSMA in prostate biopsy specimens of BPH patients after phytotherapeutic compound administration.

Molecular and immunohistochemical analysis were well related to morphological pictures. In fact, in prostate from BPH patients, the histopathological analysis showed a typical pattern of the disease: the glandular structures were lined by tall columnar or pseudo-stratified epithelium. On the contrary, in BPH patients treated with Ser-Se-Ly for 3 months, the glandular epithelium was formed by a single layer of cuboidal cells.

Overall, these findings point out the key role played by NAIP and survivin in the positive regulation of apoptosis during BPH; they also indicate that, in the context of translational medicine, these members of IAPs family could represent an interesting and innovative target in prostate homeostasis.

However, some limitations of the study must be pointed out. First, we did not perform (Enzyme-Linked ImmunoSorbent Assay (ELISA) test to verify the absolute variation of IAPs between groups. Second, the short follow-up (3 months) may be insufficient to test the efficacy of a treatment that may require more time to act. Third, the mean PSA levels at baseline were greater when compared to the data from literature. However, asymptomatic prostatitis is a factor contributing to PSA elevation due to the increase of proinflammatory cytokines and inflammatory cells that can be greater in tissues. Finally, the sample size power was based on a percentage reduction of surviving, rather than a variation between a continuous variable. Nevertheless, the strength of this study lies in the fact that this is the first time that the IAPs system in BPH patients treated with Ser-Se-Ly has been analyzed.

## 4. Materials and Methods

The study was prospectively conducted between January 2013 and January 2015, on patients affected by LUTS due to bladder outlet obstruction, secondary to clinical BPH. The trial was conducted in concordance with the ethical principles of the Declaration of Helsinki and was approved by the local Ethical Committee (ISRCTN78639965, 9 October 2011). Patients with serum prostate-specific antigen (PSA) (≥4.0 and ≤10 ng/mL) were included in this study, and underwent 12 cores prostate biopsy (with two needle biopsies collected from the transition zone) under local anesthesia. Exclusion criteria were patients with bladder cancer/prostate cancer, diabetes mellitus, neurogenic disorders, severe liver disease, history of postural hypotension, symptomatic urinary tract infections (UTI), those taking anti-androgens or antidepressants (neuroleptics, anticholinergics), previous medical or surgical treatment, and patients with catheter or an episode of acute retention of urine in the last 4 weeks.

One hundred patients with histological results of BPH were finally enrolled. Ninety consecutive patients met inclusion criteria, while ten patients were excluded for different reasons: five were lost at follow-up, three refused to participate, and two showed decompensated diabetes mellitus. Participants were randomized into two treatment arms: group A (placebo group; 45 subjects) and group B (Ser 320 mg, Ly and Se (Profluss^®^) 1 tablet per day; 45 subjects); which were examined at time 0 and after 3 months. After 3 months, patients underwent 12 cores prostatic biopsy (with two needle biopsies collected from the transition zone) under local anesthesia. All cores were collected to evaluate survivin, NAIP and caspase-3 expression, histological patterns, and prostate specific antigen (PSA) and prostate specific membrane antigen (PSMA) immunoexpression, to verify their changes from baseline to 3 months.

### 4.1. Western Blot Analysis

Total cellular proteins were extracted in a lysis buffer (25 mMTris–HCl pH 7.4, 1.0 mM ethylene glycol tetraacetic acid (EGTA), 1.0 mM ethylenediaminetetraacetic acid (EDTA), 0.5 mM phenyl methylsulphonyl fluoride, protease, and phosphatase inhibitors (100 mM Na_3_VO_4_, aprotinin, leupeptin, pepstatin at 10 µg/mL each)). The cell lysate was centrifugated at 13,000 rpm for 15 min; the supernatant was used for protein concentration determination by Bio-Rad protein assay (Bio-Rad, Richmond, CA, USA), and then diluted with Laemmli buffer. Protein samples were denatured in reducing buffer (62 mMTris pH 6.8, 10% glycerol, 2% SDS, 5% b-mercaptoethanol, 0.003% bromophenol blue), separated by electrophoresis on SDS polyacrylamide gel (6% or 10%) for approximately 1 h, and transferred to a polyvinylidene difluoride (PVDF) membrane in a transfer buffer [39 mM glycine, 48 mMTris–HCl (pH 8.3), 20% methanol] at 200 mA for 1 h. The membranes were blocked with 5% non-fat dry milk in TBS-0.1% Tween-20 for 1 h at room temperature, washed three times for 10 min each in TBS-0.1% Tween-20, and incubated with a primary antibody for survivin (Cell Signaling Technology, Beverly, MA, USA), NAIP, and caspase-3 (Abcam, Cambridge, UK) overnight at 4 °C. The day after, the membranes were washed three times for 10 min in TBS-0.1% Tween-20, and were incubated with a specific peroxidase-conjugated secondary antibody (KPL, Milford, MA, USA) for 1 h at room temperature. Following other washings, the membranes were analyzed by enhanced chemiluminescence (KPL, Milford, MA, USA). Protein signals were quantified by scanning densitometry using a bio-image analysis system (C-DiGit Blot Scanner with Image Studio software, version 5.0) and the results were expressed as relative integrated intensity compared to controls. β-Actin (Cell Signaling Technology, Beverly, MA, USA) was used to confirm equal protein loading and blotting.

### 4.2. Light Microscopy (LM)

The biopsies were fixed in 4% paraformaldehyde in 0.2 M phosphate buffer saline (PBS), dehydrated in graded ethanol, cleared in xylene and embedded in paraffin. 5 µm sections were stained with hematoxylin and eosin (H&E) and were photographed with a Zeiss Primo Star (Carl Zeiss Inc., Oberkochen, Germany) light microscope.

### 4.3. Immunohistochemistry for PSA and PSMA

Histological sections (4 µm thick) were cleared in xylene and rehydrated in graded ethanol. For antigen retrieval, the sections were incubated with 0.1 M citrate buffer (pH 6), and endogenous peroxidase blocking was performed with 3% H_2_O_2_ in PBS. Primary antibodies (PSA: clone ER.PR8, Dako, Glostrup, Denmark; PSMA: Cell Signaling Technology, Danvers, MA, USA) were applied at a dilution of 1:100 overnight at 4 °C in a moisturized chamber; the day after, the secondary antibody (Vectastain anti-rabbit, Vector, Burlingame, CA, USA) was added and the reaction visualized with 3,3′-Diaminobenzidine (DAB) (Sigma-Aldrich, Milan, Italy). Nuclear counterstaining was performed with Mayer’s haematoxylin. Negative control slices were tested using PBS instead of primary antibody. The immunostained sections were then evaluated by light microscopy. The percentage of stained cells (area of staining positivity, ASP) was graded as follows: 0 (no staining), 1 (>0%–5%), 2 (>5%–50%), and 3 (>50%). The intensity of staining (IS) (weak = 1; moderate = 2; strong = 3) was also taken into consideration. Successively, a semi-quantitative intensity-distribution (ID) score was calculated for each case by multiplying the values of the ASP and the IS, as previously described [34].

### 4.4. Statistical Analysis

Based on previous pilot study, this randomized study was designed to enroll 45 patients per group (assuming a 10% dropout rate), using two-sided of a level of 0.05 with 90% power, and assuming a decrease of 80% of survivin between groups. Values are provided as median (interquartile range—IQR). The statistical significance of differences among groups was performed with Mann-Whitney comparison tests. A *p*-value ≤ 0.05 was considered statistically significant.

## 5. Conclusions

In conclusion, a modulation of the apoptosis pathway may represent an effective strategy for BPH control. In fact, the association Ser-Se-Ly proved to be effective in promoting apoptosis in BPH patients. This report is the first to demonstrate that IAPs molecular signaling pathway is a potential target of the association Ser-Se-Ly to limit the prostatic growth in BPH patients. Our results add to a growing body of literature indicating a beneficial effect of the association Ser-Se-Ly for managing BPH. We propose that this association induces a positive modulation of the apoptosis pathways, which, in combination with other mechanisms, produces a decrease in the cellular proliferation; thus slowing down the progression of BPH.

## Figures and Tables

**Table 1:** Baseline characteristics of patients.

Parameters	Group A (*n* = 45)	Group B (*n* = 45)
Age, median (IQR)	65 (55–75)	65 (56–76)
PSA (ng/mL)	6.0 (4.2–7.8)	6.2 (4.3–8.0)
Prostate volume (cc), median (IQR)	43 (25–60)	45 (25–65)
Qmax (mL/s), median (IQR)	12 (5.4–15.0)	12.0 (6.0–14.5)
PVR (cc), median (IQR)	30 (10.0–80.0)	32 (10.0–75.0)
IPSS, median (IQR)	18 (12–28)	20.0 (12.0–29.0)
